# Factors Influencing Anti-Malarial Prophylaxis and Iron Supplementation Non-Compliance among Pregnant Women in Simiyu Region, Tanzania

**DOI:** 10.3390/ijerph13070626

**Published:** 2016-06-23

**Authors:** Benatus Sambili, Ronald Kimambo, Yun Peng, Elison Ishunga, Edna Matasha, Godfrey Matumu, Rita Noronha, David P. Ngilangwa

**Affiliations:** Amref Health Africa Tanzania, P.O. Box 2773, Dar es Salaam, Tanzania; benatus.sambili@amref.org (B.S.); ronkimambo@gmail.com (R.K.); peng.y.annie@gmail.com (Y.P.); elisonheslon@yahoo.com (E.I.); eahptanzania@gmail.com (E.M.); godfrey.matumu@amref.org (G.M.); rita.noronha@amref.org (R.N.)

**Keywords:** anti-malarial prophylaxis, iron supplementation, non-compliance, pregnant women, Tanzania

## Abstract

Malaria and iron-deficient anemia during pregnancy pose considerable risks for the mother and newborn. Intermittent Preventive Treatment during pregnancy with sulphadoxine-pyrimethamine (IPTp-SP) and iron supplement to prevent anemia to all pregnant women receiving antenatal care (ANC) services is highly recommended. However, their compliance remains low. This study aimed at identifying factors influencing non-compliance of medications among pregnant women. A descriptive cross-sectional study was conducted in Simiyu region in northwest Tanzania using a structured questionnaire to collect data from 430 women who were pregnant or gave birth 12 months prior to data collection. Data were analyzed using non-parametric statistical analysis with STATA 10. Overall, 284 (66%) and 195 (45%) of interviewed women received IPTp-SP and iron supplementation during their ANC visits, respectively. The majority (85%) of women whom received medications were aware if they had received IPTp-SP or iron supplementation. Of those received IPTp-SP, only 11% took all the three doses, while the remaining 89% took only two doses or one dose. For women who received iron supplementation, 29% reported that they did not take any dose at all. Reasons given for not complying with regiments included not liking the medications and disapproval from male partners. Our findings suggest that IPTp-SP and iron supplement compliance among pregnant women in Simiyu region is low. Intensification of community education, further qualitative research and administration of medication through directly-observed therapy (DOT) are recommended to address the problem.

## 1. Introduction

Anemia, defined as insufficient number of red blood cells in circulation or as hemoglobin level below a healthy threshold which is 12.1 to 15.1 g per deciliter (g/dL) [1,2]. It affects people across socio-economic background [1]. Globally, 1.6 billion people are affected by anemia. However, the problem is more serious among pregnant affecting 41.8% and 47.4% of them, respectively women and young children whereby 41.8% and 47.4% respectively are anemic [3]. The most common cause of anemia is iron deficiency [4]. In 2010, almost 53% of pregnant women and 39% of women who were neither pregnant nor breastfeeding were anemic in Tanzania [5]. Pregnancy heightens risk of anemia as the woman’s body transfers more of its iron to the growing fetus [6]. Iron-deficiency anemia can lead to miscarriage, still birth, premature birth, and low birth weight in babies [4]. It is also a significant contributor to maternal death, possibly because anemic women are more likely to hemorrhage [7]. It is estimated that up to 20% of maternal deaths in the world are due to the mother being severely anemic [8].

Most developing countries have policies in place to provide pregnant women with iron supplement, given in pill form [9]. In Tanzania, health policies are in line with those World Health Organization (WHO) which recommends that pregnant women, especially those at risk of being malnourished, to take 60 mg of iron daily [10]. However, one of the national surveys showed that only 10% of women took iron supplements for a minimum of 90 days, while 52% took iron supplement for 60 days or less, and 38% did not take iron supplement at all [11].

Malaria is another significant threat to pregnant women’s health. Each year, around 23 million women conceive in malaria-endemic zones in Africa [12]. Malaria contracted during pregnancy increases the mother’s risk of becoming anemic, infecting the fetus with congenital malaria, and delivering low birth weight (LBW) babies [13]. One estimate suggests that malaria during pregnancy is a factor contributing between 75,000 and 200,000 infant deaths per year [14]. Another study in northern Tanzania estimates that 20% of maternal deaths are due to malaria infection during pregnancy [15]. Tanzania’s national malaria guideline recommends that the Intermittent Preventative Treatment (IPTp) of malaria, whereby pregnant women receive two doses of sulphadoxine*-*pyrimethamine (SP) during the second and the third trimesters, as part of their routine antenatal care. However, The Tanzania Demography and Health Survey (TDHS) of 2010 estimates that only 27% of pregnant women visiting ANC clinics received a full course of IPT [5].

These interventions—SP tablets for malaria prevention and iron supplement for anemia prevention—are well-established in medical guidelines, but they are only effective if women are compliant in taking the medications [13]. Few studies across Africa have documented low compliance of IPTp [4,13,16]. It is a concern to the public health and medical communities, not only because of the negative consequences to the patient, but also because of the waste of the program resources. One study in Malawi estimated that if women’s compliance in taking SP increased from 36% to 80%, the cost of preventing infection in one person would be reduced by 90% [17].

The medical community has traditionally believed that low-compliance was caused by a drug’s side effects, or due to patient’s ignorance or lack of personal responsibility. However, more recent African-based studies have shown that women have complex reasons for not taking the medication prescribed, and these reasons are rooted in socio-cultural context that deeply shapes one’s behavior [16]. As well, there is now wider acknowledgement of environmental factors outside of the patient’s control that influences non-compliance. Examples of such environmental factors include inconsistent supply or poor quality of drugs in clinics, unfriendly staff attitudes and difficulty accessing services [4].

While the issues of SP and iron supplement non-compliance in pregnant women have been studied in some of the Sub Saharan African countries, there are few studies conducted in Tanzania [4,9,16,17]. This paper presents the factors influencing anti-malarial prophylaxis and iron supplementation non-compliance among pregnant women in Simiyu region, in northwestern Tanzania. It attempts to provide an overall picture of pregnant women’s compliance with the above two mentioned medications, by collecting data on environmental as well as individual behavioral factors. The study explores women’s ANC attendance, whether they were prescribed SP and iron supplements during their visit, whether they took them as advised and their reasons for not complying, in order to compare the evidence from Tanzania and elsewhere. The data included here were derived from a larger baseline assessment conducted by Amref Health Africa as part of its programme aimed at improving maternal, newborn and child health status in the region. 

## 2. Methods

### 2.1. Study Design and Settings 

**Figure 1:**
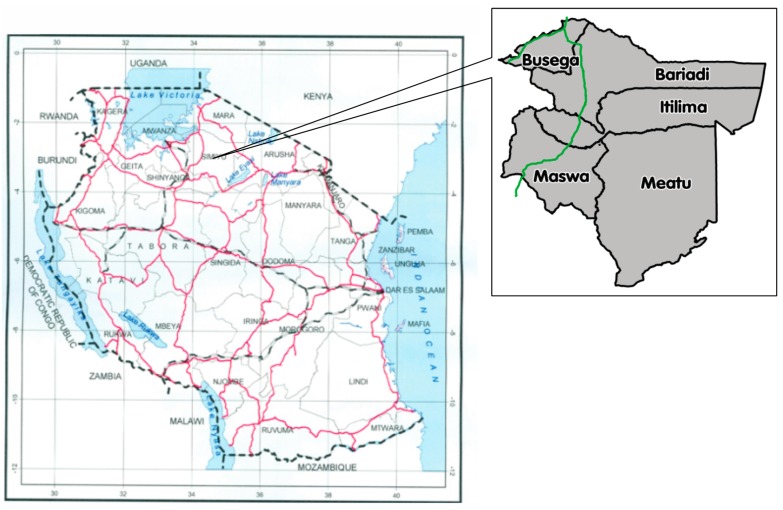
Map of Tanzania showing the study area [20].

This is a descriptive cross sectional study conducted using the quantitative data. The study was conducted in the Simiyu region in northwest Tanzania (Figure 1). Simiyu is one of 30 administrative regions in Tanzania. It is a new region carved out from the Shinyanga and Mwanza regions. The region is made up of five districts. Four out of the five districts (Bariadi, Itilima, Maswa and Meatu) were part of Shinyanga region, while the fifth one, Busega, was part of Mwanza region. The region occupies a total area of 25,212 km^2^. According to the 2012 national census the estimated population is 1,584,057. The region is among the fifteen most densely populated in the country [18]. From the outlined demographic profile it may be deduced that malnutrition in children and women is likely to be a major problem in Simiyu. Being a new region, Simiyu does not feature in the TDHS 2010 [5]. The Simiyu region provides indicative data reflecting poor outcomes for maternal, newborn and child health in the areas. Findings from a previous national representative survey showed that the prevalence of teenage pregnancies was at 11.7%; and it ranked lowest in contraceptive use at 7.5% [19]. In addition, Health Information Management Systems (HIMS) recorded 1696 deaths among children under five, mostly due to preventable conditions like malaria, anaemia, perinatal conditions, diarrhoea and acute respiratory infections. The number of infants who died in the area as recorded in health facilities was >1000, of which 547 were still-births; 311 due to asphyxia, and a shocking 799 deaths were due to malaria malaria.

Figure 1 below is the map of Tanzania, on the left it depicts clearly Simiyu region’s administrative boundaries while the small map on the right depicts Simiyu regions’s five districts.

### 2.2. Study Population and Sampling

Selection of villages was based on the health facility service area. In each of the five districts, random sampling was used to select health facilities. First, a list of all health facilities in each district was compiled and stratified based on location (rural and urban). In each stratum, one health facility was randomly selected, to comprise one urban site and one rural site from each district. In total, 10 health facilities were selected from the region. Following this, a list of all sub-villages within the government prescribed service area of the selected health facility was generated and three sub-villages per health facility were randomly selected from the list. A total of 30 sub-villages were thus selected for the study. 

After consultation with the sub-village leader, a list of all households with children under five years of age was compiled. Ten households with children less than five years of age in the household were selected randomly from the list. At the household level, the head of the household was asked to list all *de facto* members (individuals who slept in the household the night before the survey day) of his/her household. Demographic data were then collected by the interviewer using the questionnaires. Each individual within the study household was assigned a unique identification number. This was done as part of the larger baseline assessment for the programme implemented by Amref Health Africa. All women aged between 15 to 49 years in the household were invited to participate in the study if they were *de facto* household members. 

### 2.3. Data Collection 

A standardized questionnaire was administered in Kiswahili (a local language which study participants were conversant with) by trained research assistants from January to February 2013. The survey tool captured women’s socio-demographic information, parity, the pregnancy outcome, whether they delivered at home or at a health facility, the number of live births and number of neonatal deaths in their pregnancy history. The survey also asked women about their ANC visit: what medication they received and what types of health education they received. A study tool was pre-tested to assess its validity and reliability for the collection of intended information. 

### 2.4. Ethical Considerations

The study was reviewed and approved by the Amref Health Africa Institutional Review Board (IRB/AMREF/2013/04/01). In addition, local government authority at regional and district levels approved the study to take place. Written informed consent was sought from all study participants.

### 2.5. Data Processing and Analysis

Answers from the questionnaire were double entered using Census and Surveys processing System (U.S. Census Bureau and Macro International, Suitland, MD, USA, 2006) and data were checked against the paper copy for consistency and accuracy. Data cleaning was also performed involving range and consistency checks, all outliers were rectified. Two levels of quality control (QC) of the data were employed. During data collection, a subset of questions was asked to 10% of the study respondents by an independent interviewer, the two responses were then compared and clarifications sought in the event of discrepancies. Lists of identification numbers to be interviewed for the 10% QC were kept by the Supervisor of the interviewers. The second level of QC was done during the data entry phase. Data were entered by two independent data entry clerks, their entry was compared and any discrepancies verified. Data were analyzed using non-parametric statistical analysis with Stata 10 (StataCorp, College Station, TX, USA).

## 3. Results

### 3.1. Socio-Demographic Characteristics of Study Participants

In total, 430 women participated in this study. About 34% of them were aged between 25 and 34 years, representing the majority of the survey respondents. This was followed by women in the age category of 15–24, which represent 33.1% of the participants. The age distribution of female respondents did not vary significantly by study districts (χ^2^ = 8.0, *p* = 0.43). Almost 39% of the women interviewed had no formal education, 16% were primary school drop-out, 35% had completed primary school and 10% had completed secondary school education (Table 1). 

The majority of women interviewed (86%) had given birth at least once in their life. This proportion was highest in Bariadi district (93%) and lowest in Meatu district (80%). The majority of women (73%) had between 3 and 5 children, this number was highest in Maswa (84%) and lowest in Bariadi district (66%). Six percent of the women reported to have lost a child immediately after delivery, and almost 11% of the women interviewed reported to have lost a child within one month of delivery. This proportion was highest in Itilima (16%) and lowest in Busega (7%) districts. 

### 3.2. Pregnancy Status and Delivery

Of the women interviewed, 39 (9%) were pregnant during the survey. Among them, almost 39% were in their first trimester, 18% in their second trimester and 44% in their third trimester. Of those who had given birth previously, 58% delivered in a health facility, 23% delivered at home and 19% delivered at the traditional birth attendant. Major reasons given for home delivery included long distance from health facilities and family traditions and having no preparations for childbirth (Table 2).

### 3.3. ANC Attendance during the Previous Pregnancy

**Figure 2:**
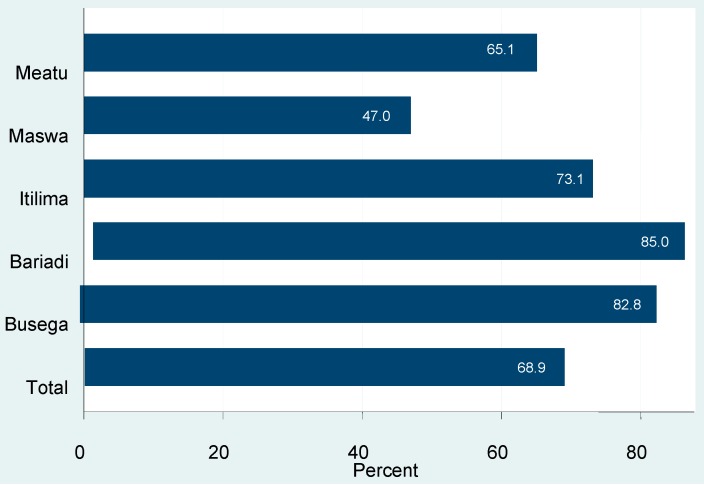
Women who attended ANC in their previous pregnancy in Simiyu region by district, in 2012.

Almost 69% of the women who were pregnant in the last 12 months visited an ANC. This proportion was highest in Bariadi (85%) and Busega districts (83%), and lowest in Maswa (47%). Only 29% of the women had attended ANC four times or more as recommended by the Ministry of Health and Social Welfare (Figure 2).

**Figure 3:**
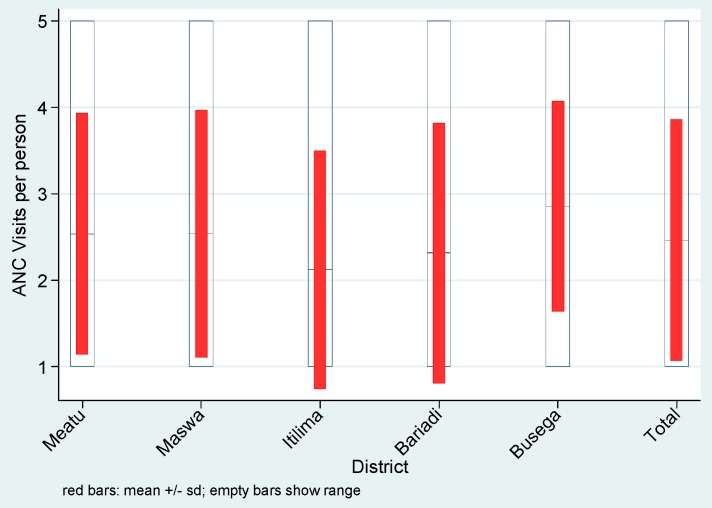
ANC visits by District in Simiyu region in 2012.

The median number of ANC visits for pregnant women in Simiyu was 2 (IQR = 1, 4). The median number of ANC visit was high in Busega (3 visits, IQR = 2, 4) compared to other districts (Figure 3).

### 3.4. Anti-Malarial Prophylaxis and Iron Tablets Medication Non-Compliance

Women who attended ANC during their previous pregnancy were asked about the preventative medications they received. Almost 66% reported that they received anti-malaria prophylaxis; this proportion was highest in Bariadi (78%) and lowest in Busega (54%). Of those who received anti-malaria prophylaxis, around 85% were aware that they received SP tablets and the rest reported that they received other forms of prophylaxis.

Women were also asked if they swallowed the medication given and only 11% reported that they swallowed all three doses, while 27% reported they swallowed two doses and 62% reported they swallowed just the first dose. The lowest rate for medication compliance was observed in Bariadi district where nearly half (48%) of the women did not swallow the tablets. Reasons given for not swallowing the tablet were as follows; 42% did not like it, 21% said that their husbands did not allow them to take. Furthermore, 20% feared that the medications might cause miscarriage (Table 3).

### 3.5. Iron Supplement and Health Education

In this study, only 45% of the pregnant women who attended ANC were given iron and folic acid supplementation. Of these, only 8% received supplements for 3 months or more and 71% reported that they took supplements given. Reasons for not complying with medication regiment include: supplements made me sick (47%), did not like them (23%), supplement gave other health problems (17%), and other reasons were they do not need them ,to avoid vomiting, thought drugs will harm unborn child or will make the baby get big (13%, Table 4).

## 4. Discussion

This study assessed women’s level of attendance for ANC clinics during their pregnancy, the dosage of iron supplements and SP tablets they received during their visit, and their compliance with the prescribed medication. The majority of women (69%) interviewed reported that they had attended an ANC clinic during their previous pregnancies. However, only 29% had attended ANC four times or more as recommended by the Ministry of Health in Tanzania. National records found that 80% of Tanzanian women attend ANC clinics at least once in their pregnancy [21]. This finding is similar to other studies conducted in Malawi and Nigeria which suggested ANCs serve an important function of reaching a large number of pregnant women with necessary medication and health education [16,22].

Such findings mean HCPs will often only have one opportunity to make a positive impression on women in order to attract them to return for follow-up care. HCPs’ attitude and service quality have been noted as one of the major reasons why women do not use ANCs. Another study in northern Tanzania which interviewed women qualitatively on their experience attending ANC clinic found that the majority of respondents complained of poor treatment from HCPs [23]. They talked about nurses scolding them or otherwise treating them like children. Shortage of staff and shortage of space mean women have to wait for a long time to be seen, and in other instances they are turned away altogether [23]. Such service problems reduce the likelihood of women consistently seeking formal healthcare during their pregnancy. In fact, there is evidence to suggest that clients value quality of care more than the distance they travel to the clinic [24]. Other studies in Tanzania have found that users’ perception of the quality of care they receive influences their health seeking behavior and treatment compliance [25]. Therefore, policy makers should think about focusing more resources toward improving infrastructure and service quality, rather than increasing the quantity of clinics in an area.

This study found that out of 228 of women who attended ANC clinics, only 66% (150) received SP tablets and only 45% (103) received iron tablets. The second figure is marginally higher than the national average (41%) for women who did not receive iron supplementation in the preceding five years [5], however both figures suggest there is still a serious problem needing to be addressed. One reason for the low level of distribution could be that HCPs’ lack of awareness of the need to prescribe these medications and supplements for pregnant women. Even though such recommendations are written in the national health policies, they may not have been disseminated as guidelines and protocols that are available in rural/remote health facilities to inform the practice of care providers.

Another reason for low level of distribution could be stock out or low supply of medication, which is a frequent occurrence in the Tanzanian healthcare system, caused by problems in the supply chain or supply management. Inconsistent supply has been cited in various other studies as a major factor affecting the patients’ likelihood to return to the clinic for follow up visits and their motivation to comply with medication when it is given [5,13,16]. A study of malaria prophylaxis programs in Zimbabwe and elsewhere and study of iron supplement programs in India both found that supply problems attributed to overall patient non-compliance and decreasing reliance on the formal medical system [17,24,26]. Therefore, we recommend the stakeholders working on ANC as well as policy makers to ensure that all respective HCPs are being educated and supervised regularly to ensure that all women who attend ANC receive antimarial. In addition, Ministry of health, Council Health Management Teams and Health facilities should work together to ensure that there is no stock out of antimalarial in health facilities.

Rural women in Tanzania on average attend ANC clinic late in their pregnancy, this often delays the administration of IPT where women are unable to receive the full two doses of the medication before they deliver. One study in Malawi found that long travelling distances, inaccessibility of facility, as well as other socio-cultural reasons influence women’s decision of whether and when to seek antenatal care [16]. Late ANC attendance, coupled with medication supply issues, act as major barriers to effective maternal malaria prevention.

The present study found evidence that there is alarmingly high number of women in the Tanzania who do not take the SP tablets prescribed to them, with only 11% of women who swallowed the two doses required as part of the IPT. The number for iron supplement non-compliance is much lower, at around 30%, but this is still a worth considering. This evidence supports findings from other studies which suggest medication non-compliance in pregnant women is a serious issue to address in developing countries [5,9,13,16,23,27,28,29]. Some of the main reasons women gave in these studies for their choice in non-compliance included: not liking the medication, fear of miscarriage and fear that medication would bring other health problems. Other studies on iron supplement compliance have found that the look and the taste of the medication highly affect women’s willingness to take them [9]. Also, a lack of accurate information and the presence of misinformation regarding the drug’s effect and potential side-effect on mother and fetus heighten fear and hampers the patient’s acceptance of the medication. A study on iron supplementation found women often believe the pills would lead to bigger babies or heavier hemorrhaging during delivery [30]. Another study in northern Tanzania found that women feared experiencing side effect in taking SP, possibly the Steven-Johnson syndrome, although this is a rare occurrence [12].

Finally, 20% of women reported husband disapproval as one of the reason for not swallowing the SP tablets. This suggests that partner’s attitude toward the medication deeply influences women’s behavior in this regard. The systematic review by Nagata, Gatti and Barg [30] has found that the support woman receives from her social network: family, neighbors, community leaders and local HCPs greatly improves her motivation to continue with medication regiment. However, lack of support or negative attitudes from those around her produces the opposite effect. This demonstrates that sensitization efforts must make special attempt to bring a woman’s family members and community leaders into the conversation, and help them become the woman’s allies in attaining optimal health for the mother and child.

Regarding the much higher adherence rate reported for iron supplements, it is possible that women perceived them more positively than they did for SP tablets. Systematic review of literature found that in Nigeria, women believe iron pills can increase blood and give strength, and are seen as beneficial to health because they are being prescribed by the HCP [30]. Another explanation for this observed trend is that patients over-report their level of adherence [5]. However, for those who did not comply, the most common reason given was that the tablet produced side effects, or made them feel sick. One study found that with education and support from HCP, most women manage to overcome the side effects and continue with their regimen, again highlighting the important the role quality of care plays in patient compliance [30]. This clearly calls for health education of both the wife and husband, we therefore recommend that at least during the first ANC visit for both partners should be available for health and nutrition education. This session should also clearly inform the couple the importance of antimalarial prophylaxis and iron supplementation, as well as address their fears and superstitions about the medications.

Our study adds to the body of knowledge on factors for non-compliance of anti-malaria prophylaxis and iron supplements among women during pregnancy. However, the data set of this study was drawn from a larger baseline study investigating many other variables. Thus there were relatively few questions relating to medication prescription during ANC visits. We were not able to explore more in-depth women’s reasons for refusing to comply with medication and how much each factor influenced their decision. Also, the cross-sectional study design provided only a snap shot in time and did not allow us to understand if women’s compliance changed over time or over the course of several pregnancies. Finally, self-reported data on medication use is one of the least reliable methods of measuring, while direct measuring such as testing physiological changes in the body are considered more accurate [5], financial and time constraints did not permit undertaking such studies. Nevertheless, our study maintained its internal and external validity throughout its implementation.

## 5. Conclusions

The findings from this study are consistent with the evidence gathered from other countries, and suggest that efforts are needed in Tanzania to address the issue of access and compliance to iron supplement and SP among pregnant women. Several key factors must be taken into consideration in such intervention efforts. The importance of regular and timely ANC clinic visit cannot be understated. Many women delay their clinic visit till the 2nd or 3rd trimester, which makes it impossible to deliver the full dosages of medication needed for her to maintain optimal health. Therefore, community level health education and sensitization efforts are needed to address the myths and misinformation circulating regarding iron supplements and SP, and to foster more receptive attitudes toward adherence in women and in their social circles. Also, we recommend the involvement of women’s partners in ANC visits however we understand that there might be challenging to get some of the partners especial in teen pregnancies. In addition, wherever feasible administration of medication through directly-observed therapy (DOT), would be another option to increase uptake of both iron supplements and SP.

## Figures and Tables

**Table 1:** Socio-demographic characteristics of participants in Smiyu region in 2013.

Background Variables	*n* = 430	%
**Age (in years)**		
15–24	142	33.1
25–34	146	34.0
≥35	142	32.9
**Education**		
No education	167	38.8
Some primary school	69	16.0
Completed primary school	150	34.9
Secondary and above	44	10.2
**District of residence**		
Bariadi	88	20.5
Busega	83	19.3
Itilima	87	20.2
Maswa	93	21.6
Meatu	79	18.4
**Ever given birth**		
No	59	13.8
Yes	369	86.2
**Number of biological children**		
1–2	128	34.7
3–5	140	37.9
≥6	101	27.4
**Living with their children**		
No	32	8.8
Yes	331	91.2
**Women whose children died within a month of delivery**		
No	383	89.1
Yes	47	10.9
**Women whose children died after delivery**		
No	406	94.4
Yes	24	5.6

**Table 2:** Pregnancy status and place of delivery among women in Simiyu region, Tanzania in 2013.

Pregnancy Variables	*n* = 430	%
**Are you pregnant now?**		
No	391	90.9
Yes	39	9.1
**Gestation age**		
1st trimester	15	38.5
2nd trimester	7	18.0
3rd trimester	17	43.6
**Place of birth of the last child**		
Home	85	23.0
Traditional birth attendant	71	19.2
Health facility	212	57.5
others	1	0.3
**Reasons for home delivery**		
Heath facility is far from home	32	37.7
I did not have money for transport	9	10.6
Services at health facility are poor	2	2.4
Others	42	49.4

**Table 3:** Anti-malaria prophylaxis and women compliance data in Simiyu region in 2013.

Anti-Malarial Variables	*n* = 228	%
**Were anti-malaria prophylaxis received during ANC visits?**		
Yes	150	65.8
No	78	34.2
**Self-reported type of anti-malaria prophylaxis received**		
*SP*	127	84.7
Other prophylaxis	23	15.3
**If received, was it swallowed?**		
Yes	100	66.7
No	50	33.3
**How many doses did you swallow?**		
One	62	62.0
Two	27	27.0
Three	11	11.0
**Why you did not swallow?**		
I did not like them	13	26.0
Fear of miscarriage	6	12.0
My husband did not allow me	10	20.0
Other reasons	21	41.0

**Table 4:** Iron supplement and health education given during ANC visits among women in Simiyu region in 2013.

Type of Service Provided	*n* = 228	%
**Was iron supplements provided during ANC visits?**		
Yes	103	45.2
No	125	54.8
**Duration iron supplements were used**		
<3 months	95	92.8
≥3 months	8	7.2
**Were iron supplements taken as advised?**		
Yes	73	70.9
No	30	29.1
**Why you did not take iron supplements as advised?**		
I did not like them	7	23.6
They made me sick	14	46.7
They caused other health problems	5	16.7
Other reasons	4	13.3
**Were the health education provided during ANC visits?**		
Yes	77	33.8
No	151	66.2
**Was nutrition education provided during ANC visits?**		
Yes	98	43
No	130	57
